# Complement Factor H Is an Early Predictive Biomarker of the Therapeutic Efficacy of Sublingual Immunotherapy for Japanese Cedar Pollinosis

**DOI:** 10.3390/pathogens11111280

**Published:** 2022-11-01

**Authors:** Riyo Yoneda, Tomohisa Iinuma, Daiju Sakurai, Junya Kurita, Tomoyuki Arai, Yuri Sonobe, Syuji Yonekura, Yoshitaka Okamoto, Toyoyuki Hanazawa

**Affiliations:** 1 Department of Otorhinolaryngology, Head and Neck Surgery, Chiba University Graduate School of Medicine, 1-8-1 Inohana, Chuo-ku, Chiba 260-8670, Japan; 2 Department of Otolaryngology, Head and Neck Surgery, Yamanashi University Hospital, 1110 Shimokato, Chuo, Yamanashi 409-3898, Japan; 3 Department of Immunology, Chiba University Graduate School of Medicine, 1-8-1 Inohana, Chuo-ku, Chiba 260-8670, Japan; 4 Chiba Rosai Hospital, 2-16 Tatsumidaihigashi, Ichihara 290-0003, Japan

**Keywords:** sublingual immunotherapy, Japanese cedar pollinosis, biomarker

## Abstract

Sublingual immunotherapy for Japanese cedar pollinosis can improve the symptoms of allergic rhinitis and modify its natural course. However, sublingual immunotherapy requires a long treatment period and some patients do not respond to treatment. In this study, we aimed to identify biomarkers that could predict the efficacy of sublingual immunotherapy at an early stage. In this study, 40 patients from phase III trials were recruited and divided into good and poor response groups. Using peripheral blood mononuclear cells from before and two months after the start of medication, microarray, discriminant analysis, and real-time polymerase chain reaction were performed to extract candidate genes that could be biomarkers. Furthermore, these genes were validated in 30 patients in general clinical practice. Complement factor H was upregulated in the good response group and downregulated in the poor response group. Complement factor H may be a useful biomarker for predicting the efficacy of sublingual immunotherapy for Japanese cedar pollinosis at early time points after treatment initiation.

## 1. Introduction

Japanese cedar pollinosis (JCP) is an allergic rhinitis unique to Japan and the number of patients continues to increase annually. The incidence of JCP in children, which was previously thought to be rare, is also becoming a problem [1,2]. As the number of JCP patients increases, it has become clear that the disease also has a significant impact on sleep, learning, and labor productivity and reduces the quality of the daily life of patients. In addition, once JCP has developed, spontaneous improvement is rare except in elderly patients, and there is a need for effective basic treatment and the prevention of its onset [3,4,5,6].

Allergen-specific immunotherapy is the only therapy that can modify the natural course of allergic rhinitis and improve its symptoms. Sublingual immunotherapy (SLIT), in which antigens are administered under the tongue, has been confirmed to be efficacious and safe. In Japan, SLIT for JCP was launched in 2014, and SLIT for mite allergic rhinitis in 2015. Currently, the Japanese guidelines for allergic rhinitis recommend SLIT for all severities of JCP [1,7].

However, SLIT must be continued for at least three years to maintain efficacy. Moreover, despite the long treatment period, treatment efficacy has been found to be poor in 20–30% of cases [6,8]. In addition, at least one treatment season is required to determine the efficacy of SLIT for JCP. It is also difficult to determine its efficacy objectively. In fact, in a double-blind, placebo-controlled Phase III trial, the proportion of patients with symptoms that improved in the first season was 61% in the actual drug group compared to 39.4% in the placebo group. SLIT is considered a treatment with a high placebo effect [6]. Patient satisfaction was high in the actual drug group, although because improvement in subjective symptoms includes a placebo effect, patients with high satisfaction due to the placebo effect may experience a relapse of symptoms after SLIT completion. Owing to the long treatment period, a high dropout rate has been reported [9]. Although several markers are associated with SLIT mechanisms, there is no consensus on suitable prognostic, predictive, or surrogate biomarker candidates for clinical responses to SLIT [10,11,12,13,14]. It is difficult to determine whether SLIT should be continued in patients who do not respond clearly in the first season; therefore, if its efficacy can be predicted earlier than the first season, it may motivate patients to continue SLIT. However, for patients with poor efficacy, alternative medicine can be suggested, which benefits patients by reducing the burden. Thus, there is a great need to identify a biomarker that can predict the efficacy of SLIT before therapy or early during therapy and establish it as an assessment method. This study aimed to identify early biomarkers of SLIT and predict their efficacy. We examined two different populations: Study 1 and Study 2. The patient population was divided into good and poor response groups according to their response to SLIT. In Study 1, blood samples from patients in a double-blind placebo-controlled trial were used. Using these samples, microarray, discriminant analysis, and real-time polymerase chain reaction (PCR) were performed to identify candidate biomarker genes. In Study 2, the candidate biomarker gene was revalidated by real-time PCR in general clinical patients.

## 2. Materials and Methods

### 2.1. Study Design

**Figure 1:**
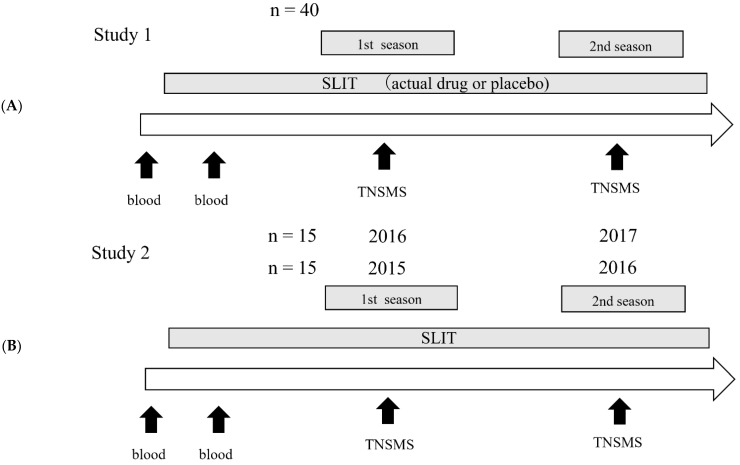
Outline of the medication and follow-up procedures in (**A**) Study 1 and (**B**) Study 2. SLIT, sublingual immunotherapy; TNSMS, Total Nasal Symptom and Medication Score.

In Study 1, 40 patients from a double-blind, placebo-controlled trial of the efficacy and safety of SLIT for JCP patients conducted from 2010 to 2012 were included in the analysis at Chiba University Hospital. Patients were treated with either the actual drug or placebo from the non-pollen season until the end of the second cedar pollen dispersal season (Figure 1A).

In Study 2, 30 patients who received SLIT for JCP from general clinical practice at Chiba University Hospital were recruited. Fifteen patients started SLIT in the non-pollen season in 2014 and continued through the cedar dispersal season in 2015 and 2016; 15 patients started SLIT in the non-pollen season in 2015 and passed the cedar dispersal season in 2016 and 2017 (Figure 1B). In both Studies 1 and 2, blood samples were collected before and two months after the initiation of medication.

The pollen counts for each year were 6537, 1256, 2910, 3304, and 1820/cm^2^ in 2011, 2012, 2015, 2016, and 2017, respectively.

Efficacy was evaluated using the Total Nasal Symptom and Medication Score (TNSMS) from the Japanese nasal allergy guidelines [1]. The TNSMS was calculated as the sum of nasal symptoms—sneezing, rhinorrhea, and nasal congestion—using a 5-point scale (0, none; 1, mild; 2, moderate; 3, severe; 4, very severe) and the drug scores of oral drugs and nasal sprays.

### 2.2. Determination of Treatment Efficacy

The good and poor response groups in Studies 1 and 2 were defined differently because the amount of cedar pollen dispersal varied from year to year.

#### 2.2.1. Study 1

The TNSMS in the peak symptom period was calculated in the first and second seasons and classified into four levels of severity according to the Japanese nasal allergy guidelines: most severe, severe, moderate, and mild. The good response group consisted of patients whose symptoms improved by two or more steps in the second season or improved to mild in the second season. The poor response group consisted of patients whose symptoms did not improve or worsen during the second season. Patients who did not meet these criteria were excluded from this study. Based on these criteria, the patients were divided into four groups: good response in the SLIT group (Act/good), poor response in the SLIT group (Act/poor), good response in the placebo group (Plc/good), and poor response in the placebo group (Plc/poor). Of the 40 patients, 7 were assigned to the Act/good group, 4 to the Act/poor group, 5 to the Plc/good group, and 7 to the Plc/poor group (Table 1).

#### 2.2.2. Study 2

As in Study 1, the TNSMS in the peak symptom period was calculated in the first and second seasons. The rate of change in TNSMS between the first and second seasons was determined, and 15 patients whose symptoms improved from the median rate of change were in the good response group and 15 patients whose symptoms did not improve were in the poor response group (Table 2).

### 2.3. Microarray

Peripheral blood mononuclear cells (PBMCs) were isolated from peripheral blood using LYMPHO SEPARATION MEDIUM (MP Biomedical, Aurora, OH, USA), as previously reported [15]. Total RNA was isolated from PBMCs, and labeled cRNA was prepared using the Quick Amp Labeling Kit (Agilent Technologies, Santa Clara, CA, USA), followed by hybridization. Microarray slides were analyzed using an Agilent DNA microarray scanner (Agilent Technologies, Santa Clara, CA, USA). The measured image data were quality-checked and quantified using Feature Extraction software (Agilent Technologies, Santa Clara, CA, USA).

Two-way analysis of variance (ANOVA) with one-factor correspondence was performed. The Tukey–Kramer method in the time-point direction and the Steel–Dwass test in the sample direction were used to identify significant differences. Variable genes with *p*-values < 0.05 were extracted by ANOVA, followed by checking for fold-change and sub-testing.

### 2.4. Real-Time PCR

RNA was reverse-transcribed to cDNA using the Superscript VILO cDNA reverse transcription kit (Life Technologies, Carlsbad, CA, USA) following the manufacturer’s instructions. TaqMan PCR primers were obtained as proprietary pre-optimized reagents (Life Technologies, Carlsbad, CA, USA), and probes were obtained from the Universal Probe Library (Roche, Basel, Switzerland). PCR primers and probes were combined with TaqMan gene expression master mix in real-time PCR reactions (7900HT Fast Real-Time PCR System; Applied Biosystems, Waltham, MA). The relative quantitation of mRNA expression was performed using the 2^∆∆Ct^ method relative to the housekeeping gene (18S rRNA).

### 2.5. Statistical Analysis

Discriminant analysis was conducted by constructing discriminant equations for each gene, using the treatment’s good or poor response as the objective variable and the numerical data before and two months after the medication started as the explanatory variables. The “qda” function in MASS, an R package, was used for analysis (R Foundation for Statistical Computing, Vienna, Austria). Posterior probabilities greater than 50% were considered correct. The Mann–Whitney U test was used to compare two groups using GraphPad Prism software (version 8.43; GraphPad Software, San Diego, CA, USA), and the significance level was set at *p* < 0.05.

## 3. Results

### 3.1. Candidate Narrowing by Microarray

**Figure 2:**
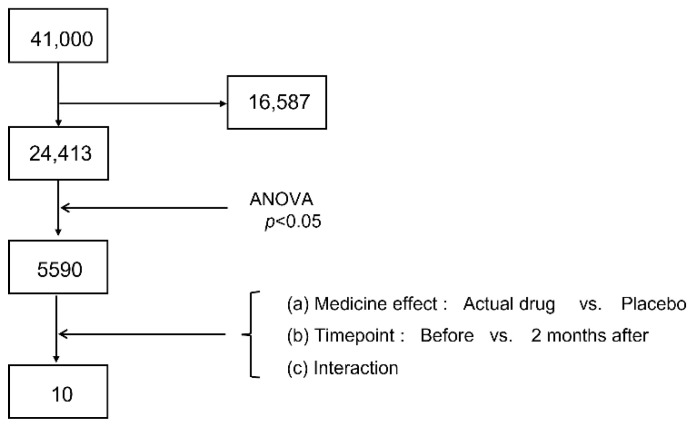
Schema of gene narrowing by microarray. ANOVA, analysis of variance.

In Study 1, candidate genes for predictive biomarkers were narrowed down by dividing them into four groups: Act/good, Act/poor, Plc/good, and Plc/poor. Of the 41,000 probes mounted on the microarray, 24,413 were selected for analysis, and the remaining probes were removed with signal values equivalent to the background (Figure 2). Among the selected probes, 5590 probes were selected as probes with a *p*-value < 0.05 by ANOVA. We compared the selected genes according to two main effects and their interaction. The main effects were (a) the medicine effect (actual drug vs. placebo) and (b) the time point (before vs. two months after the medication started), and the third factor was (c) their interaction with the main effect. Candidate genes were selected if they varied with a *p*-value of < 0.05, and if their behavior differed only in the Act/good group; that is, if they were upregulated or downregulated only in the Act/good group. Three genes (*CFH*, *PRKCB*, and *SEPT7P2*) were selected for the comparison between the SLIT and placebo groups. *GOLIM4* was selected as a candidate gene for comparison between before and two months after the initiation of medication. Six genes were selected for the interaction between the two factors: *EIF2D*, *FSCN1*, *DTX3*, *TTC39C*, *TUBB3*, and *ATP8B3*. Ten genes were selected as candidate predictive biomarkers (Table 3).

### 3.2. Candidate Narrowing by Real-Time PCR

**Figure 3:**
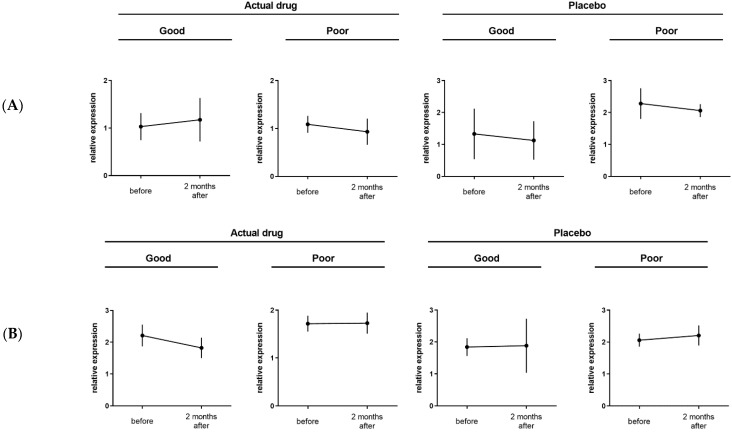
Relative expression of (**A**) *CFH* and (**B**) *GOLIM4* selected by real-time polymerase chain reaction.

Real-time PCR was performed for ten candidate genes that were selected by microarray in Study 1 using PBMCs collected before and two months after the start of treatment. Gene expression changes before and two months after the start of medication were analyzed in each of the four groups: Act/good, Act/poor, Plc/good, and Plc/poor. Among the ten candidate biomarker genes, *CFH* and *GOLIM4* were selected as genes whose variation showed the same behavior as the microarray results (Figure 3): *CFH* in the Act/good group was upregulated from before to two months after the medication started, and *GOLIM4* in the Act/good group was downregulated from before to two months after the medication started. Due to the small number of cases, there was no significant difference in gene expression levels before and two months after the initiation of medication.

### 3.3. Evaluation of Prediction Accuracy by Discriminant Analysis

**Figure 4:**
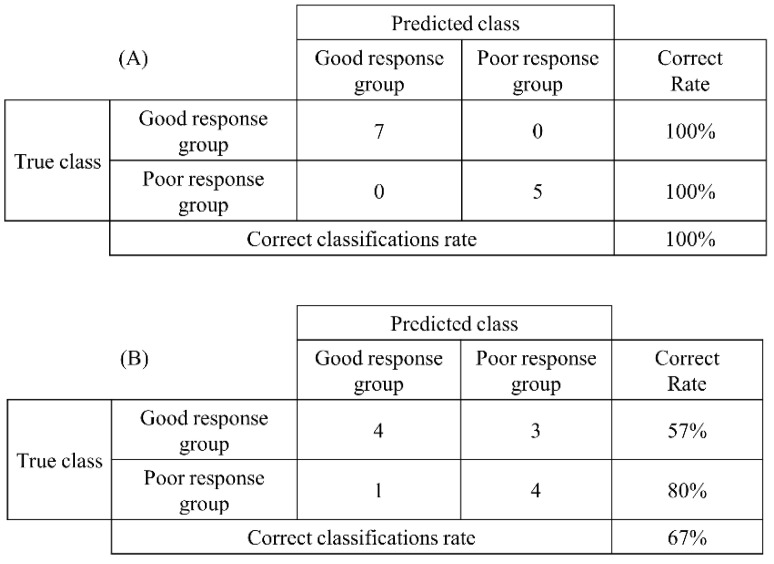
Discriminant analysis of real-time polymerase chain reaction results. (**A**) *CFH* and (**B**) *GOLIM4*. A high percentage of correct classifications reflects a good result.

The accuracy of *CFH* and *GOLIM4,* determined using real-time PCR in Study 1, as predictive biomarkers for the efficacy of SLIT was analyzed using discriminant analysis. Analysis was performed on the Act/good and Act/poor groups. The results of the discriminant analysis are presented in a confusion matrix. The non-linear discriminant analysis of the changes in *CFH* expression from before to two months after the medication started showed a 100% correct rate in both the Act/good and Act/poor groups. The correct classification rate was 100%. The non-linear discriminant analysis of the changes in *GOLIM4* expression from before to two months after the medication started showed a 57% correct rate in the Act/good group and an 80% correct rate in the Act/poor group. The correct classification rate was 67% (Figure 4).

These results suggest that *CFH* can predict the efficacy of SLIT with a high degree of accuracy by measuring gene expression changes before and two months after the start of treatment.

### 3.4. Efficacy of CFH as a Biomarker

**Figure 5:**
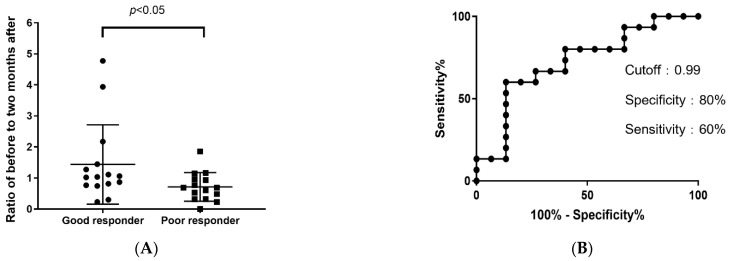
Variation in *CFH* expression. (**A**) The ratio of *CFH* expression before to two months after the start of medication between the good and poor response groups. (**B**) Receiver operating characteristic curve for the rate of change in *CFH*.

*CFH* expression before and two months after the start of medication was confirmed in Study 2. Real-time PCR was performed using PBMCs before and two months after the initiation of medication. The *CFH* expression two months after the start of medication was compared to that before the medication started. The ratio of *CFH* expression before the start of treatment to two months after the start of treatment was obtained. The ratio was set to 1 when there was no change between before and two months after the start of medication. The ratio was greater than 1 when *CFH* expression increased from before to two months after the start of medication and was less than 1 when decreased. The mean values of the ratio in the good and poor response groups were 1.43 (standard deviation (SD) = 1.23) and 0.71 (SD = 0.44), respectively. T-test results showed a significant difference between the two groups (t (28) = 2.06, *p* < 0.05). *CFH* increased in the good response group and decreased in the poor response group. This is consistent with the results of Study 1 (Figure 5A). Using a receiver operating characteristic curve and setting the cutoff value to 0.99, the specificity and sensitivity were 80% and 60%, respectively (Figure 5B).

## 4. Discussion

Several studies have predicted the efficacy of SLIT; however, there is still no established predictive model. We investigated the factors that could predict SLIT efficacy at an early stage. A microarray, real-time PCR, and discriminant analysis were used to narrow down candidate genes in PBMCs from a double-blind, placebo-controlled trial. Based on the microarray results, ten genes were identified as candidate predictive biomarkers. Two genes were selected that behaved similarly to the microarray results obtained by real-time PCR: *CFH* and *GOLIM4*. These two genes, *CFH* and *GOLIM4*, encode complement factor H (CFH) and Golgi integral membrane protein 4 (GOLIM4), respectively. The accuracy of these two genes as predictive biomarkers was examined using discriminant analysis, and a high correct classification rate was obtained for *CFH*. Real-time PCR was performed on samples from general clinical patients and showed the same behavior as the microarray. In both studies, *CFH* was found to increase in the good response group and decrease in the poor response group when comparing levels before and two months after the medication started. The cutoff value for the rate of change in *CFH* was 0.99, with a sensitivity of 60% and specificity of 80%. *CFH* expression has been suggested as a potential predictive biomarker for the therapeutic efficacy of SLIT for JCP.

CFH is the first protein regulated in the alternative pathway of the complement system. CFH is a single-chain glycoprotein that binds to the active forms of C3 and C3b and promotes the dissociation and deactivation of the C3 convertase C3bBb or C3bBbP (P: properdin) in the alternative pathway when bound to C3b [16]. In addition, it controls C5 convertase activity by competitively inhibiting the binding of C3b to C5. CFH acts as a cofactor for factor I, which cleaves C3b to inactive iC3b. C3a and C5a are involved in allergic inflammation, such as by anaphylatoxins, in the migration of eosinophils and mast cells, and C5a participates in the migration of macrophages, neutrophils, and basophils. When these cells are activated, leukotrienes, histamines, cytokines, and chemokines are released. Complement activation affects the allergic state via the complement activation pathway, including the classical and alternative pathways [17,18]. There are reports that serum C3a and C5a levels are significantly decreased by the long-term administration of SLIT [19,20,21]. Since CFH acts as an inhibitor of the alternative pathway, C3a and C5a are thought to decrease when CFH is upregulated in the good response group. In this study, the change in CFH was attributed to SLIT. Elevated CFH causes C3a and C5a to decrease and, as a result, inhibits the allergic reaction and suppresses allergens other than cedar. However, since this is an antigen-specific immunotherapy, it is unlikely that the observed *CFH* expression changes in PBMCs affect overall immunity. Although its effectiveness as a biomarker is expected, its relevance as a mechanism of action remains unknown. Further studies are required to clarify the mechanism of action and to determine if CFH can be used as a valuable biomarker for other sublingual immunotherapies.

The classification of the good and poor response groups in these studies has limitations. In Study 1, the amount of cedar pollen dispersal varied from year to year, with large amounts in the first season and small amounts in the second season; therefore, the classification was based on changes in symptoms from the first to the second season. In Study 2, there was no significant change in the amount of dispersal over the three years; therefore, we classified whether symptoms improved from the first to the second season. In both Studies 1 and 2, we compared the change in symptoms between the first and second seasons to classify the good and poor response groups. However, patients whose symptoms improved immediately after the medication started were already classified as mild in the first season and did not improve thereafter. These cases might have been lost to follow-up in the poor response group.

We determined the ratios of *CFH* expression before and two months after the start of treatment and compared them between the good and poor response groups. However, the SDs were large; thus, in most cases, the ratios were approximately 1. Among the individual patients, those with even a slight increase in CFH expression were more likely to benefit from treatment, whereas those with decreased *CFH* expression experienced a limited effect.

The cutoff value was 0.99, with a sensitivity of 60% and specificity of 80%, making it a useful predictive biomarker of the good response group, although less accurate as a predictive biomarker of the poor response group. A good response motivates patients to continue treatment even if they do not clearly respond in the first season. For the poor response group, it is possible to suggest the discontinuation of SLIT and transition to alternative treatment; however, the results of this study were not precise enough to exclude the poor response group. We will continue to investigate the significance of this predictive biomarker not only in the good response group but also in the poor response group, based on prospective comparative studies.

## 5. Conclusions

We searched for biomarkers that could predict the efficacy of SLIT for JCP at an early stage of therapy. *CFH* expression was found to increase in the good response group and decrease in the poor response group early after medication initiation, indicating that *CFH* may be a biomarker that can predict the efficacy of SLIT early during treatment.

## Figures and Tables

**Table 1:** Patient characteristics in Study 1.

Act/Good	Sex	Age	sIgE	First Season Severity	Second Season Severity
	F	60	50.9	moderate	mild
	M	61	31.4	very severe	moderate
	M	52	20.4	very severe	moderate
	F	43	16.0	severe	mild
	F	47	8.7	moderate	mild
	F	29	8.5	moderate	mild
	F	48	6.7	severe	mild
**Act/Poor**	**Sex**	**Age**	**sIgE**	**First Season** **Severity**	**Second Season** **Severity**
	F	55	100.0	moderate	moderate
	F	42	10.5	moderate	moderate
	M	48	8.9	moderate	moderate
	F	51	5.2	moderate	severe
**Plc/Good**	**Sex**	**Age**	**sIgE**	**First Season** **Severity**	**Second Season** **Severity**
	F	49	52.8	very severe	moderate
	M	43	19.5	very severe	moderate
	F	37	8.8	severe	mild
	F	48	8.8	severe	mild
	F	41	5.8	moderate	mild
**Plc/Poor**	**Sex**	**Age**	**sIgE**	**First Season** **Severity**	**Second Season** **Severity**
	M	58	38.1	moderate	severe
	F	52	27.1	moderate	moderate
	M	43	16.5	severe	severe
	M	58	15.6	moderate	moderate
	F	49	8.2	moderate	moderate
	F	28	5.2	moderate	moderate
	F	26	3.8	moderate	moderate

F, female; M, male; Act/good, good response to SLIT; Act/poor, poor response to SLIT; Plc/good, good response to placebo; Plc/poor, poor response to placebo; SLIT, sublingual immunotherapy; sIgE, specific IgE.

**Table 2:** Patient characteristics in Study 2.

Good	Sex	Age	sIgE	First Season TNSMS	Second Season TNSMS
	F	35	96.9	7.58	5.10
	M	43	85.7	5.94	4.35
	F	41	65.9	7.68	5.00
	F	62	58.4	5.71	4.32
	M	34	39.3	8.32	6.48
	F	18	37.8	8.43	5.05
	F	41	34.3	6.84	2.76
	M	21	32.6	3.00	1.43
	F	25	20.7	8.89	3.00
	F	63	19.2	4.43	3.26
	F	49	17.8	5.10	3.11
	F	61	10.1	7.16	5.67
	M	61	7.3	3.32	2.86
	M	27	5.2	5.05	3.71
	F	55	3.9	2.38	1.53
**Poor**	**Sex**	**Age**	**sIgE**	**First Season TNSMS**	**Second Season TNSMS**
	F	50	39.3	3.67	3.21
	M	24	21.3	3.11	3.38
	M	28	15.3	4.19	5.84
	F	30	11.1	3.16	3.00
	F	54	10.5	6.05	7.53
	F	42	8.6	8.29	8.16
	F	52	8.0	5.00	8.05
	M	49	7.2	2.22	2.53
	F	57	7.1	4.16	3.95
	F	56	6.8	6.20	6.17
	F	45	5.9	2.81	3.42
	F	45	4.8	4.19	5.16
	M	48	3.5	2.70	3.26
	F	60	1.8	1.74	2.62
	M	31	1.1	3.21	3.10

F, female; M, male; Good, good response group; Poor, poor response group; sIgE, specific IgE; TNSMS, Total Nasal Symptom and Medication Score.

**Table 3:** Ten genes selected by microarray.

		ANOVA			Lower Rank Test							
		Medication	Time	Interaction	Act/Good		Act/Poor		Plc/Good		Plc/Poor	
Gene Symbol	ANOVA Category	*p*-Value	*p*-Value	*p*-Value	FC	Reg.	FC	Reg.	FC	Reg.	FC	Reg.
*CFH*	a	0.0239	0.7449	0.4867	1.23	up	1.08	down	1.10	down	1.02	down
*PRKCB*	a	0.0321	0.6997	0.1785	1.14	down	1.06	up	1.04	up	1.02	up
*SEPT7P2*	a	0.0237	0.8003	0.2965	1.14	down	1.06	up	1.03	up	1.04	up
*GOLIM4*	b	0.5854	0.0151	0.2764	1.50	down	1.03	up	1.14	up	1.24	up
*EIF2D*	c	0.5891	0.4864	0.0091	1.08	up	1.00	down	1.03	down	1.10	down
*FSCN1*	c	0.5431	0.8282	0.0415	1.83	up	1.70	down	1.06	down	1.10	down
*DTX3*	c	0.4475	0.0793	0.0097	1.13	up	1.32	down	1.01	down	1.17	down
*TTC39C*	c	0.2651	0.2308	0.0278	1.12	up	1.18	down	1.03	down	1.12	down
*TUBB3*	c	0.5786	0.2574	0.0058	1.30	down	1.14	up	1.00	up	1.52	up
*ATP8B3*	c	0.3515	0.1176	0.0114	1.18	down	1.13	up	1.17	up	1.23	up

Act/good, good response to SLIT; Act/poor, poor response to SLIT; Plc/good, good response to placebo; Plc/poor, poor response to placebo; FC, fold change; Reg., regulation; SLIT, sublingual immunotherapy; ANOVA, analysis of variance.

## Data Availability

The data that support the findings of this study are available on request from the corresponding author. The data are not publicly available due to patents.
